# Phonon-based partition of (ZnSe-like) semiconductor mixed crystals on approach to their pressure-induced structural transition

**DOI:** 10.1038/s41598-020-76509-0

**Published:** 2020-11-13

**Authors:** M. B. Shoker, Olivier Pagès, V. J. B. Torres, A. Polian, J.-P. Itié, G. K. Pradhan, C. Narayana, M. N. Rao, R. Rao, C. Gardiennet, G. Kervern, K. Strzałkowski, F. Firszt

**Affiliations:** 1 Université de Lorraine, LCP-A2MC, ER 4632, 57000 Metz, France; 2 Departamento de Fisica and I3N, Universidade de Aveiro, 3810-193 Aveiro, Portugal; 3 Institut de Minéralogie, de Physique des Matériaux et de Cosmochimie, Sorbonne Université—UMR CNRS 7590, 75005 Paris, France; 4 Synchrotron SOLEIL, L’Orme Des Merisiers Saint-Aubin, BP 48, 91192 Gif-sur-Yvette Cedex, France; 5 Department of Physics, School of Applied Sciences, KIIT Deemed to be University, Bhubaneswar, Odisha 751024 India; 6 Jawaharlal Nehru Centre for Advanced Scientific Research (JNCASR), Jakkur P.O., Bangalore, 560064 India; 7 Solid State Physics Division, Bhabha Atomic Research Centre, Mumbai, 400085 India; 8 Laboratoire de Cristallographie, Résonance Magnétique Et Modélisations, UMR 7036, Université de Lorraine, 54506 Vandoeuvre-lès-Nancy, France; 9 Institute of Physics, N. Copernicus University, 87-100, Toruń, Poland

**Keywords:** Materials science, Physics

## Abstract

The generic 1-bond → 2-mode “percolation-type” Raman signal inherent to the short bond of common A_1−x_B_x_C semiconductor mixed crystals with zincblende (cubic) structure is exploited as a sensitive “mesoscope” to explore how various ZnSe-based systems engage their pressure-induced structural transition (to rock-salt) at the sub-macroscopic scale—with a focus on Zn_1−x_Cd_x_Se. The Raman doublet, that distinguishes between the AC- and BC-like environments of the short bond, is reactive to pressure: either it closes (Zn_1−x_Be_x_Se, ZnSe_1−x_S_x_) or it opens (Zn_1−x_Cd_x_Se), depending on the hardening rates of the two environments under pressure. A partition of II–VI and III–V mixed crystals is accordingly outlined. Of special interest is the “closure” case, in which the system resonantly stabilizes *ante* transition at its “exceptional point” corresponding to a virtual decoupling, by overdamping, of the two oscillators forming the Raman doublet. At this limit, the chain-connected bonds of the short species (taken as the minor one) freeze along the chain into a rigid backbone. This reveals a capacity behind alloying to reduce the thermal conductivity as well as the thermalization rate of photo-generated electrons.

## Introduction

A_1−x_B_x_C semiconductor mixed crystals with zincblende structure^1^ are benchmark systems for the experimental study of the chemical disorder due to alloying, which relates to the percolation site theory^2–4^. The C-invariant and (A,B)-substituting sublattices intercalate through a tetrahedral bonding, resulting in two bond species (A–C and B–C) arranged with maximal (cubic) symmetry. This forms the most “simple” three-dimensional (3D) disordered system of chemical bonds one can imagine, comparable to an ideal object, which can be experimentally tested and confronted with models ran at the ultimate atom scale. The simplicity gives grounds for hope to solve certain critical issues behind alloying. One refers to the nature of the A ↔ B atom substitution^5^, as to whether this is ideally random, or not. Another one, tackled in this work, is to elucidate how lattice-supported complex media engage their pressure-induced structural transition at the local scale^6^, this being already an issue for the pure compounds^7^. Yet in order to address experimentally the raised issues, one not only needs a suitable system as described above but also a local probe, such as the bond force constant, as conveniently measured at the laboratory scale by Raman scattering.

The historical models used for the discussion of the Raman spectra of A_1−x_B_x_C zincblende mixed crystals, namely the modified-random-element-isodisplacement (MREI) and the cluster ones (both summarized in Ref.^8^), are not well suited to tackle the raised issues, for different reasons detailed elsewhere^9^. In brief, the MREI model is blind to the local environment of a bond, by construction. As for the cluster model, to our view it suffers from a conceptual bias that was shown to generate a misleading insight into the nature of the atom substitution. Moreover, both models are based on the virtual crystal approximation in which the A and B substituents are replaced by virtual A_1−x_B_x_ atoms with physical properties averaged over the A and B ones depending on the composition x. By doing so the virtual crystal approximation virtually restores a perfect chemical/structural order, which comes to view a A_1−x_B_x_C ternary mixed crystal at the macroscopic scale in terms of a pseudo-binary (A_1−x_B_x_)C compound. Somehow, this denies the essence of substitution, leaving the impression that the semiconductor mixed crystals are saved from disorder, and are thus special among complex media.

Over the past two decades we introduced an alternative so-called percolation model that views an A_1−x_B_x_C zincblende ternary at the mesoscopic scale in terms of an AC/BC-like composite^9^. A given bond vibrates at different frequencies in the AC- and BC-like regions giving rise to a generic bimodal Raman signal *per* bond (1-bond → 2-mode). This is especially clear for the short bond (say, e.g., B–C) that usually involves the substituent with a small covalent radius. As such, the short bond has more room to distort than the long one, and hence is the one that mostly accommodates the local strain due to the contrast in the A–C and B–C bond length/stiffness. This generates significant variation in the (B–C) bond force constant from site to site in the crystal depending on the local AC- or BC-like environment, with concomitant impact on the (B–C) Raman frequency, being accordingly diversified.

Just like its MREI and cluster predecessors, the percolation model is a phenomenological one in which the lattice dynamics is grasped within the one-dimension (1D) paradigm. The justification is that, as an optical method, Raman scattering can only detect long-wavelength ($\lambda \to \infty$, $q$ = 2 $\pi {\lambda }^{-1}\to$0) lattice vibrations^10^. On such length scale the information is averaged over several crystal unit cells so that there is no point in trying to spot an atom in the real (3D) lattice. For the sake of consistency, the AC- and BC-like environments of a bond are likewise described at 1D, which, for the minor species of the crystal at least (say, e.g., B–C), comes to distinguish between isolated bonds (dispersed inside the AC-like region) and self-connected ones (forming the BC-like region). In fact, a singularity is expected in the Raman frequency ($\omega$) on crossing the bond percolation threshold—in echo to a universal bond length anomaly^11^, i.e., ~ 19 at.% in the zincblende structure, at which critical composition the self-connection becomes suddenly infinite, a pure statistical effect of the random A ↔ B substitution^2^. More generally, in the percolation scheme the bond environments are defined up to second-neighbors at most. This is consistent with a basic feature of the bond charge model used to describe the phonon dispersion of semiconductor compounds (like AC and BC) with diamond and zincblende structures that phonons are essentially a matter of short range interactions^12,13^.

So far, the percolation scheme was applied to different mixed crystals with zincblende, wurtzite and diamond structures^14^ suggesting its universal character. Apparently, only wavelength-resolved vibration spectroscopies such as optical methods ($\lambda \to \infty$)—covering Raman scattering and infrared absorption^15^—and inelastic neutron scattering^16^ are sensitive to the local environment of a bond as formalized within the percolation scheme. Advanced temperature-dependent extended-X-ray-fine-structure-absorption measurements, while offering a useful overview of the entire lattice dynamics averaged over all possible wavelengths in one shot^17,18^, fail to do so.

Seen from the angle of the percolation model, Raman scattering breaks new ground for studying mixed crystals. For instance, the first issue raised above has been solved recently: when exploited within the percolation scheme, the Raman spectra of zincblende^19^ as well as diamond^20^ mixed crystals can be used to shed light on the nature of the atom substitution (random vs. clustering/anticlustering) on a quantitative basis (using an ad hoc order parameter—see below). In this work, we address the second issue and test the Raman doublet of the short bond as a sensitive chemical probe to “see” how mixed crystals engage their pressure-induced structural transition at the mesoscopic scale.

Preliminary Raman studies have been conducted in this spirit on Zn_1−x_Be_x_Se^21^ ($x$ ≤ 0.52) and ZnSe_1−x_S_x_^22^ ($x$ = 0.32), though without being able, yet, to understand the observed phenomena, listed below.

(i) The Be–Se (Zn_1−x_Be_x_Se) and Zn–S (ZnSe_1−x_S_x_) Raman doublets close under pressure, due to a progressive convergence of the lower “mode 2” onto the upper “mode 1”.
(ii) During the convergence process, mode 2 gradually collapses down to full extinction on crossing mode 1, apparently due to a dead loss of oscillator strength.
(iii) The actual crossing occurs around the same critical pressure ${P}_{c}$ at any $x$ value, falling close to the pressure-induced zincblende ↔ rock-salt structural transition of pure ZnSe.
(iv) Beyond ${P}_{c}$ only mode 1 survives; mode 2 “freezes”, testified by ab initio calculations.
  Features (i–iv), originally evidenced with Zn_1−x_Be_x_Se, were qualitatively attributed to the large contrast in the pressure-induced structural transitions of ZnSe (to the rock-salt structure^23^, at ${P}_{ZnSe}$ ~ 13 GPa) and BeSe (to the NiAs structure^23^, at ${P}_{BeSe}$ ~ 56 GPa). This seemed coherent with an ab initio trend that the Ga-P doublet of GaAs_1−x_P_x_—characterized by nearly identical pressure transitions of its parent compounds^23^ (~ 15 ± 3 GPa, zincblende → *Cmcm*)—remains quasi stable under pressure^21^. However, this was a wrong track since features (i-iv) lately repeated with the Zn–S doublet of ZnSe_1−x_S_x_^22^ even though the alleged contrast is suppressed in this case (ZnS transforms to rock-salt^23^ at ~ 13 GPa, as ZnSe). Since then, we fall short of an adequate explanation for any of the features (i–iv)^22^.
  In this work a deeper insight into the origin of (i–iv) is searched for by extending our high-pressure Raman study of ZnSe-based mixed crystals to Zn_1−x_Cd_x_Se taken in its zincblende structure (x < 0.3, the structure is wurtzite otherwise) using a single crystal. The 3-mode {1 × (Cd–Se),2 × (Zn–Se)} Raman behavior of Zn_1−x_Cd_x_Se at ambient pressure has been clarified recently^19^, a prerequisite to its high-pressure study. As features (i–iv) are not x-dependent, for the current experimental case studies we focus on Zn_0.83_Cd_0.17_Se whose Raman behavior at ambient pressure was studied in detail. Zn_0.83_Cd_0.17_Se is further interesting in that its optical band gap is nearly resonant with the green laser line used to excite the Raman spectra^1^. This offers a chance to play with the gap-related singularity in the dispersion of the refractive index in view to access the phonon-polaritons besides the conventional phonons (a similar approach with Zn_1−x_Mg_x_Se^14^ proved to be much rewarding in this respect), hence offering a Raman overview. Generally, the phonon-polaritons propagating in the volume of mixed crystals remain unexplored experimentally—not to mention under high pressure, apart from our own recent studies^14,19,22^ (and Refs. therein).
  The as-completed series of ZnSe-based systems encompasses a broad range of contrasts in bond physical properties.
(v) The Be–Se (0.420), Zn–S (0.764) and Cd–Se (0.841) bond ionicities are smaller, similar and larger, respectively, than that of Zn–Se (0.740)^24^. Therefore, the Be and Cd incorporations stiffen and soften the ZnSe lattice, respectively, whereas the S incorporation is neutral with this respect. The zincblende ↔ rock-salt transition pressures are correspondingly larger, smaller and comparable to ${P}_{ZnSe}$^23^.
(vi) The Zn–Se bond stands out among the II-VI's in that its capacity to stiffen under pressure hits the lowest level, judged by the minimal volume derivative of its bond ionicity ($d{f}_{i}^{*}/dlnV$=0.127), that makes roughly half the value for Zn–S and Cd–Se, and one sixth as much as the Be–Se one^24^.
(vii) The three systems cover various percolation-type Raman patterns, i.e., with well-separated (~ 200 cm^−1^, Zn_1−x_Be_x_Se^21^), close (~ 50 cm^−1^, ZnSe_1−x_S_x_^22^) and degenerate (~ 0 cm^−1^, Zn_1−x_Cd_x_Se^19^) AC-singlet and BC-doublet—to a point, in the latter case, that the spacing is larger for the doublet ($\delta$ ~ 20 cm^−1^) than between the doublet and the singlet ($\Delta$ ~ 10 cm^−1^) – (see, e.g., Supplementary Fig. S6b).
(viii) Zn–Se is either the “passive” (AC-singlet, Zn_1−x_Be_x_Se and ZnSe_1−x_S_x_) or the “active” (BC-doublet, Zn_x_Cd_1−x_Se) bond; the active bond is sensitive to its local environment either up to first- (Zn_1−x_Be_x_Se) or second-neighbors (ZnSe_1−x_S_x_, Zn_1−x_Cd_x_Se), and the active bond is either the minor (Be–Se, Zn–S) or the dominant (Zn–Se of Zn_1−x_Cd_x_Se) species^19,21,22^.

We hope that such variety of contrasts (v–viii) will help to get sufficient hindsight—that was sorely lacking so far—to elucidate features (i–iv). Such forward step is needed prior to validating the percolation-type Raman doublet of a bond as a useful “mesoscope” for study of semiconductor mixed crystals under pressure.

**Figure 1:**
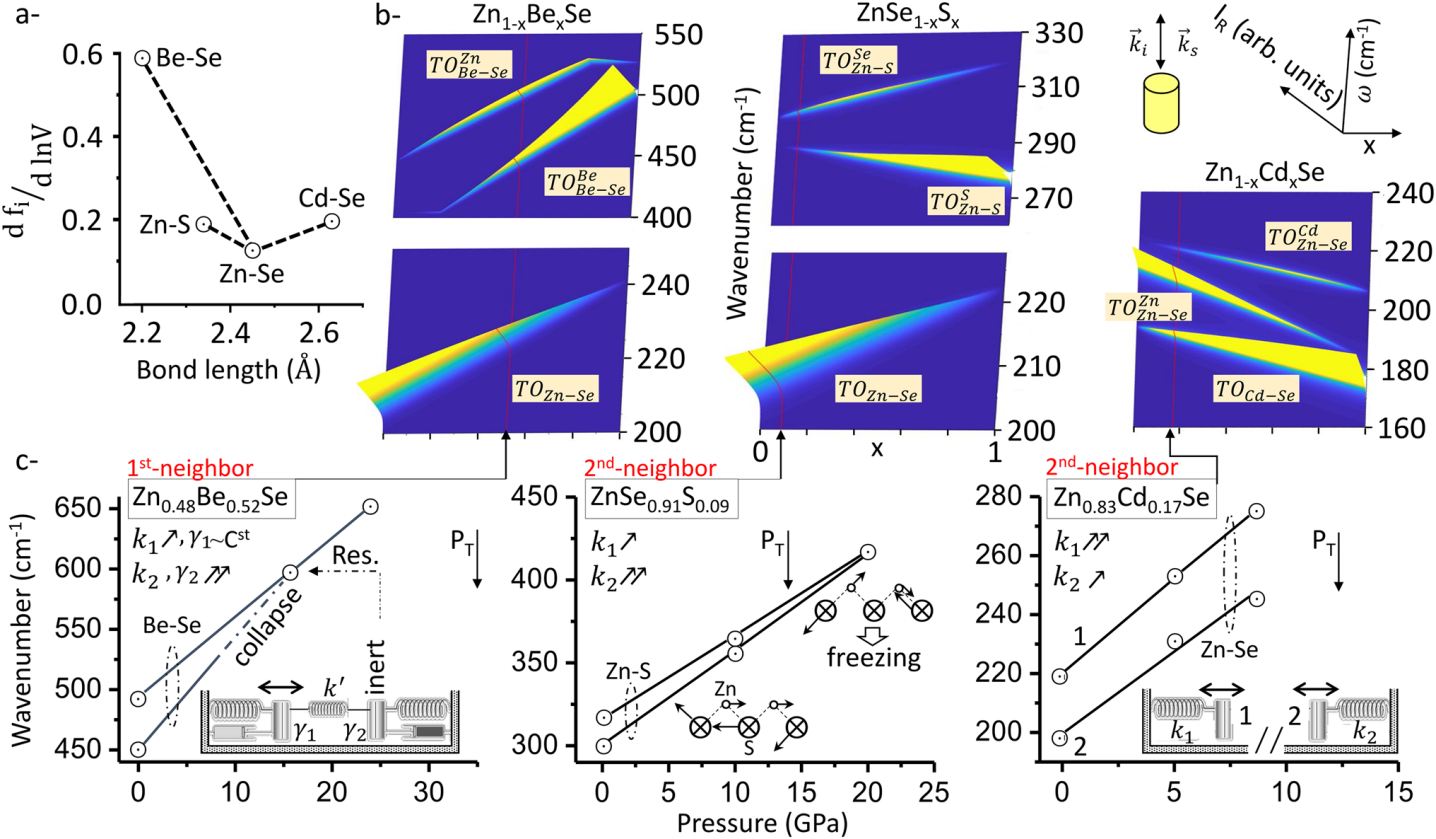
Pressure dependence of the percolation-type (purely-mechanical) TO Raman doublets of various ZnSe-based mixed crystals. (**a**) Bond length and volume dependence of bond ionicity for the constituting species of the studied mixed crystals (indicated via dashed lines)—taken from Ref.^24^, helping visualize which is the short bond (X-axis) and how its percolation-type Raman doublet changes (closure *vs*. opening) with pressure (Y-axis, see text). (**b**) Theoretical composition dependence of the Raman intensities of the Zn_1−x_Be_x_Se, ZnSe_1−x_S_x_ and Zn_1−x_Cd_x_Se TO (purely-mechanical) triplets, as apparent at 0 GPa in a backscattering Raman experiment (as sketched out). (**c**) Pressure dependence of the corresponding Be–Se (this work), Zn–S (Ref.^22^) and Zn–Se (this work) Raman doublets (dashed ovals)—reflecting sensitivity of bond vibrations up to first- or second neighbors (as specified)—at selected compositions. In the closure case (Zn_1−x_Be_x_Se, ZnSe_1−x_S_x_), the freezing of the lower oscillator—due to vibrations of self-connected bonds along the chain (central panel)—at the resonance (Res.) leads to a Raman extinction (collapse) transposing to inertia at 1D (left panel). In the opening case (Zn_1−x_Cd_x_Se), the oscillators remain independent at any pressure (right panel). The pressure dependencies of the bond force constants (${k}_{i}$) and vibration dampings (${\gamma }_{i}$)—governing the closure/opening and collapse processes of each system, respectively (see text)—are schematically indicated (using single or double arrows), together with the critical zincblende → rock-salt pressure transition (${P}_{T}$).

An overview of the pressure dependence of various Raman doublets for the current series of ZnSe-based systems, useful to fix ideas, is sketched out in Fig. 1. This reveals in advance the primary outcome of this work that the Zn–Se doublet (Zn_1−x_Cd_x_Se) opens under pressure, contrary to the Be–Se (Zn_1−x_Be_x_Se) and Zn–S (ZnSe_1−x_S_x_) ones, that close^21,22^. We detail below how the opening trend for Zn_1−x_Cd_x_Se was evidenced combining experimental and ab initio Raman insights into its polar and non-polar modes, respectively. Also, the variety of trends in Fig. 1 appears to be sufficient to trace the origin of the pressure-induced closure/opening of a percolation-type Raman doublet.

To complete the picture, the disconcerting features (i–iv) in the closure case are re-examined within a basic model of coupled/damped harmonic oscillators defined at 1D^25,26^—for the sake of consistency with the 1D-percolation scheme, focusing on Zn_~0.5_Be_~0.5_Se as a case study. A pivotal feature in this model is the so-called *exceptional point*^25,26^, characterized by a perfect balance between gain (mechanical coupling in this case) and loss (due to overdamping). Such singular points are being actively sought in optics and photonics as the source of exotic behaviors^27^, and also in phononics, notably in view to minimize the thermal conductivity in semiconductor-based devices^28,29^ (achieved through nanoarchitecturing in the cited works).

## Results and discussion

By placing the study (at 17 at.% Cd) close to the Cd-Se bond percolation threshold (i.e., ${x}_{p}$ ~ 19 at.% Cd, in case of a random substitution), we can be assured of a top-diversified mesostructure—thus a representative one, i.e., with maximum variety in the topology of the minor (Cd–Se) species: isolated as well as self-connected Cd–Se bonds are expected to coexist in significant proportions, forming, in the latter case, small as well as large (nearly infinite, on the verge of percolation) clusters, also in significant proportions^2^.

Now, Zn_1−x_Cd_x_Se is prone to clustering on approach to its composition-induced zincblende → wurtzite structural transition^19^ ($x$ ~ 0.3), which might significantly impact its mesostructure, especially at compositions near the Cd–Se bond percolation threshold as in the present case. A Raman insight into the clustering rate $\kappa$ of Zn_1−x_Cd_x_Se with moderate-to-large Cd content—on a scale of 0 (random substitution) to 1 (full clustering, i.e., phase separation)—using the terminology of the $\kappa$-based formalism developed within the cluster model^8^—has earlier been obtained within the percolation scheme based on a description of Zn_1−x_Cd_x_Se in terms of a CdSe/ZnSe-like “composite” (see above). Such Raman insight operated at the “mesoscopic” scale, leading to $\kappa$ ~ 0.5, was independently supported by ab initio calculations^19^. A more refined “microscopic” insight relying on a description of the current Zn_0.83_Cd_0.17_Se crystal in terms of five elementary Se-centered 3D-tetrahedron units with (Zn,Cd) at the vertices is currently gained by performing ^77^Se solid-state nuclear magnetic resonance (NMR) measurements on a powdered sample—along the approach earlier used with Zn_1−x_Cd_x_Te^30^, leading to $\kappa$ ~ 0.12. The latter value is only used to fix ideas since the $\kappa$-based formalism eventually appears to be non-transferable to the NMR data (Supplementary Section I).

A subsequent statistical analysis of the (Cd,Zn)–arrangement in large (10 × 10 × 10) A_x_B_1−x_C zincblende supercells reveals that the minor Cd–Se bonds percolate with the same probability in large ($x$ = 0.17, $\kappa$ ~ 0.5)-clustered and ($x$ = 0.19, $\kappa$ ~ 0)-random supercells, meaning that the bond percolation is hastened by clustering (Supplementary Section I). However, as $\kappa$ = 0.5 seems to be an upper estimate for Zn_0.83_Cd_0.17_Se (compare the Raman vs. NMR insights), we can safely state that our crystal has not yet crossed the Cd–Se bond percolation threshold. Hence, its mesostructure presumably consists of a CdSe-like dispersion embedded in a Swiss cheese-like ZnSe-like matrix (and not of two finely interwined CdSe- and ZnSe-like treelike 3D-continua).

The limit for the planned high-pressure Raman study of Zn_0.83_Cd_0.17_Se is the zincblende → rock-salt (fourfold → sixfold coordination) structural transition^23^, identified at P_CdZnSe_~ 12 GPa by high-pressure X-ray diffraction. Representative diffractograms *ante* and *post* transition in the upstroke (pressure increase) and downstroke (pressure decrease) regimes are shown in the Supplementary Section I. Remarkably, the composition dependence of the Zn_1−x_Cd_x_Se (x < 0.4) bulk modulus B_0_(x) at ambient pressure derived from the X-ray data exhibits a percolation-type singularity at 17 at.% Cd. This offers an insight into the mechanical properties of the studied Zn_0.83_Cd_0.17_Se mixed crystal at the macroscopic scale besides that achieved hereafter at the mesoscopic scale by Raman scattering.

**Figure 2:**
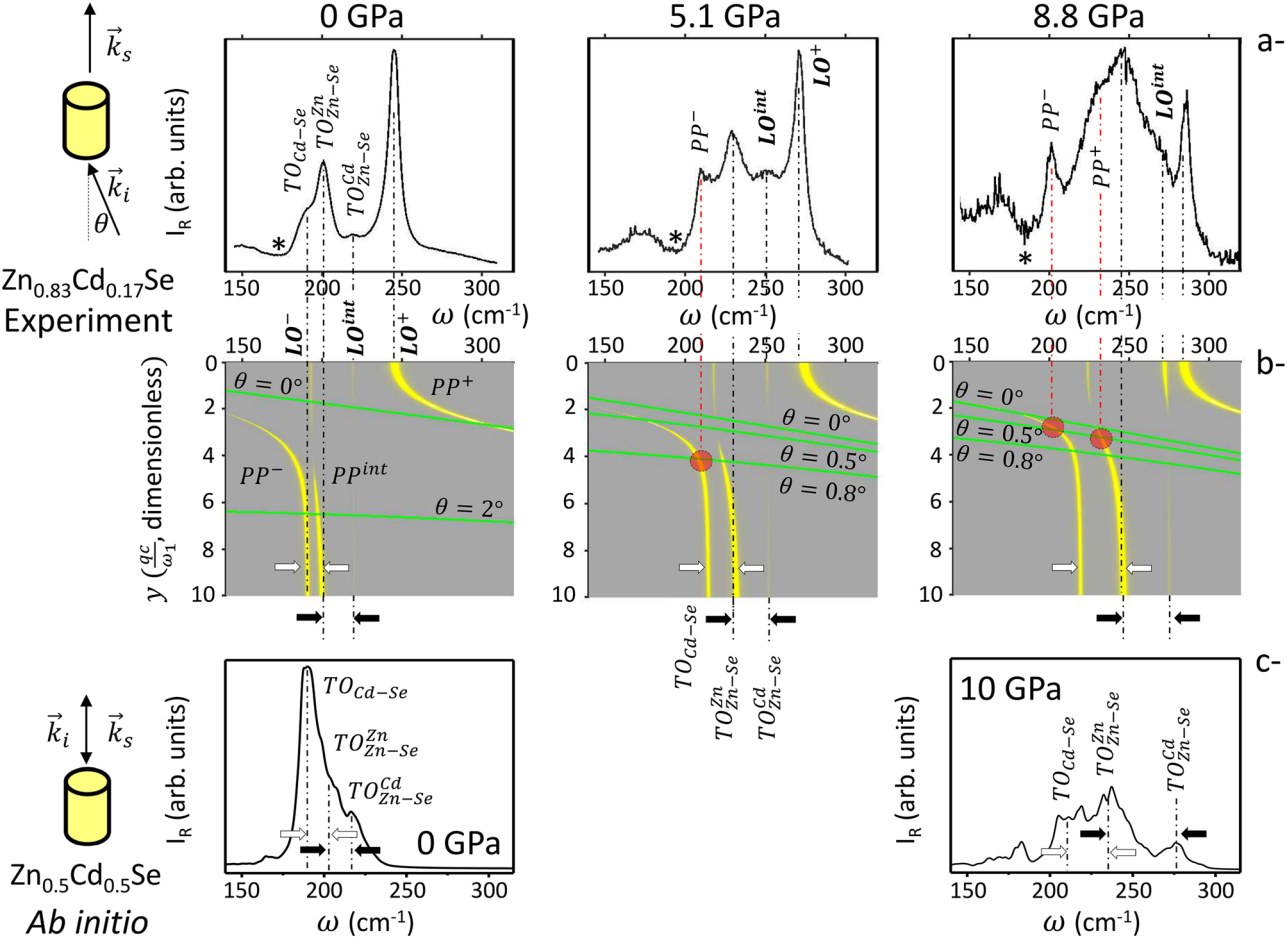
Zn_1−x_Cd_x_Se high-pressure Raman spectra. (**a**) Zn_0.83_Cd_0.17_Se near-forward Raman spectra at selected pressures—the star marks a Fano-type antiresonance. (**b**) Corresponding Raman scan lines (oblique lines) superimposed onto the relevant phonon-polariton dispersions (curves) including the Raman intensities (thickness of curves). The crossing points are emphasized (circles). (**c**) Ab initio TO (purely-mechanical) Raman spectra of a 216-atom Zn_0.5_Cd_0.5_Se disordered cubic-supercell at ambient and high pressures. Paired arrows indicate changes in frequency gaps between the Cd–Se singlet and the Zn–Se doublet (hollow) and within the Zn–Se doublet (filled) with pressure.

A selection of pressure-reversible backward (reflection-like) and forward (transmission-like) Raman spectra taken at (nearly) the same spot at low (a, ~ 0 GPa), intermediate (b, ~ 5 GPa) and high (c, ~ 9 GPa < ${P}_{CdZnSe}$) pressures through/onto the (110)-faces of a tiny Zn_0.83_Cd_0.17_Se single crystal inserted in a diamond anvil cell is shown in Fig. 2 (more spectra are shown in Supplementary Section I together with additional ones taken on a powder—Fig. S6). Such scattering geometries probe the transverse optic (TO) modes of a zincblende crystal in their purely-mechanical (PM-TO strictly speaking, but abbreviated TO hereafter) and phonon-polariton (PP) regimes^10,31^, respectively. Though the longitudinal optic (LO) modes are theoretically forbidden^31^, they also show up due to multi-reflection of the laser beam between parallel crystal faces^32^, completing an overview into the Zn_0.83_Cd_0.17_Se optic modes.

The optic modes are linked to each other: the TO and LO modes mark the asymptotic limits of the “$\omega$ vs. $q$” PP dispersion far off the center $\Gamma$ ($q$ = 0) of the Brillouin zone (being clear that as an optical technique Raman scattering operates close to $\Gamma$ anyway) and near $\Gamma$, respectively^10,31–33^. The PP dispersion (curves) and Raman intensities (thickness of curves) calculated for Zn_0.83_Cd_0.17_Se by using the generic expression of the Raman cross section given in Ref.^19^ (details are given in the Supplementary Section I) are shown in Fig. 2.

We are mostly interested in the pressure dependence of the ${TO}_{Zn{-}Se}^{Zn}-{TO}_{Zn{-}Se}^{Cd}$ doublet, the subscript specifying the bond vibration and the superscript the local environment. The focus on the TOs is justified because, generally, being non polar (purely-mechanical) vibrations they hardly couple and thus reflect the intrinsic phonon pattern at a given pressure. However, a direct experimental TO insight (as with Zn_1−x_Be_x_Se^21^) is difficult in the case of Zn_1−x_Cd_x_Se because its three-mode TO pattern is so compact (see, e.g., Fig. S6b)—to a point that Zn_1−x_Cd_x_Se was long considered to exhibit the ultimate one-mode (2-bond → 1-phonon) behavior in its Raman spectra^19^. Further the ${TO}_{Zn{-}Se}^{Cd}$ Raman mode is hardly discernible (Fig. 1) due to a non-favorable sharing of the available Zn–Se available oscillator strength between the two Zn–Se submodes at x ~ 0.17^19^. Last, on its sensitive high frequency side the TO signal is screened by the LO modes that show up strongly.

In view of such drawbacks with the TO modes we are forced to proceed with the polar PP (as with ZnSe_1−x_S_x_^22^) and LO modes, with a difficulty that they couple via their macroscopic transverse ${\overrightarrow{E}}_{T}(q)$ and longitudinal ${\overrightarrow{E}}_{L}$ electric fields, respectively. Due to the ${\overrightarrow{E}}_{T,L}$-coupling, neither a specific bond nor a specific environment can be assigned to the PPs and LOs. Therefore, these modes are simply labeled as {${PP}^{-}$, ${PP}^{int}$, ${PP}^{+}$} and {${LO}^{-}$, ${LO}^{int}$, ${LO}^{+}$} in ascending frequency (lower, intermediate, upper). Generally, the ${\overrightarrow{E}}_{T}(q)$- and ${\overrightarrow{E}}_{L}$-couplings channel the available (Cd–Se and Zn–Se) oscillator strength towards low and high frequencies, respectively^22^. Hence the PPs and the LOs are naturally well suited to investigate the compact Zn_1−x_Cd_x_Se TO pattern on its low- (CdSe-like) and high-frequency (ZnSe-like) sides, respectively.

At (nearly) ambient pressure, the situation is as follows (Fig. 2): the {${TO}_{Cd{-}Se}$, ${TO}_{Zn{-}Se}^{Zn}$, ${TO}_{Zn{-}Se}^{Cd}$} modes of Zn_0.83_Cd_0.17_Se show up at {~ 190, ~ 200 and ~ 220} cm^−1^, with their LO replicas at {~ 190, ~ 220 and ~ 245} cm^−1^. The PPs are not visible, because, as soon as they depart from their native TOs, they interfere destructively with a two-phonon continuum involving transverse acoustic modes from the Brillouin zone edge (2 × TA) that emerges nearby^19^—as independently observed with Zn_1−x_Be_x_Se^21^ and ZnSe_1−x_S_x_^22^. Under pressure, the 2 × TA zone-edge band softens (shifts to low frequency), in contrast with all zone-center ($\Gamma$-like) optic modes that harden (shift upward)^34^—a direct ab initio insight is provided as Supplementary information (Fig. S9). This opens a path for PP detection at minimal scattering angle—see “Methods”. Only ${PP}^{-}$ is observed at ~ 5 GPa (close to ${TO}_{Zn{-}Se}^{Zn}$), whereas both ${PP}^{-}$ (emerging well beneath ${TO}_{Zn{-}Se}^{Zn}$ then) and ${PP}^{int}$ come about at ~ 9 GPa. The increased PP diversity can be explained only if the native ${TO}_{Cd{-}Se}$ behind ${PP}^{-}$ breaks away from the native ${TO}_{Zn{-}Se}^{Zn}-{TO}_{Zn{-}Se}^{Cd}$ doublet of ${PP}^{int}$ under pressure (see hollow arrows in Fig. 2).

An indirect insight into the remaining upper/minor ${TO}_{Zn{-}Se}^{Cd}$ end of the Zn–Se doublet is achieved via its (quasi) degenerate ${LO}^{int}$ mode. At ambient pressure, ${LO}^{int}$ emerges as a residual feature slightly remote from the mid-[${TO}_{Zn{-}Se}^{Zn}-{LO}^{+}$] band on the TO-side^19^. By increasing pressure, ${LO}^{int}$ is shifted away from ${TO}_{Zn{-}Se}^{Cd}$ towards ${LO}^{+}$ and strengthens until arriving at quasi intensity matching with ${LO}^{+}$ at ~ 9 GPa (judging by the areas of the Raman peaks). The pressure-induced upward-shift/strengthening of ${LO}^{int}$ are intrinsic to cubic Zn_1−x_Cd_x_Se since they are also visible in the LO-like Raman spectra of disordered and semi-ordered (cubic) thin films (~ 45 at.% Cd)^35^. Their combination cannot be merely fortuitous, suggesting a common origin. This is searched for by trying a blind test on the pressure dependence of the underlying ${TO}_{Zn{-}Se}^{Cd}$ frequency behind ${LO}^{int}$, with several options: the (${TO}_{Zn{-}Se}^{Zn}-{TO}_{Zn{-}Se}^{Cd}$) doublet closes (scenario 1), remains stable (scenario 2), or widens (scenario 3) under pressure. There is no need to speculate on the ${TO}_{Zn{-}Se}^{Zn}$ frequency that is readily accessible at any pressure. Both the upward-shift/strengthening of ${LO}^{int}$ under pressure nicely fit into scenario 3 (see filled arrows in Fig. 2), whereas scenarios 1 and 2 fail to generate any of those trends (Fig. S8).

In brief, the experimental PP and LO Raman insights on each side of the compact TO pattern of Zn_0.83_Cd_0.17_Se converge to reveal that the {${TO}_{Cd{-}Se}$, ${TO}_{Zn{-}Se}^{Zn}$, ${TO}_{Zn{-}Se}^{Cd}$} triplet splits off under pressure. This applies in particular to the Zn–Se doublet (Zn_1−x_Cd_x_Se)—of central interest, hence contrasting with the Zn–S (ZnSe_1−x_S_x_) and Be–Se (Zn_1−x_Be_x_Se) doublets, which are closing under pressure. Independent support to the overall splitting arises from ab initio calculation of the high-pressure pure-TO Raman spectra related to a large (216-atom) disordered ($\kappa$ ~ 0) Zn_0.5_Cd_0.5_Se cubic-supercell (see “Methods”) corresponding to well-resolved Cd–Se and Zn–Se Raman signals (Fig. 2, bottom). Additional ab initio insight into the lattice dynamics (depending on composition and pressure) and the lattice relaxation of Zn_1−x_Cd_x_Se is provided in the Supplementary Section I.

Out of the listed contrasts (v–viii), only that related to (vi) $d{f}_{i}^{*}/dlnV$ can explain the pressure-induced closure/opening of the Raman doublet of the short bond depending on the system. Consider, e.g., the Zn–Se doublet of Zn_1−x_Cd_x_Se that distinguishes between Zn–Se TO vibrations in homo (ZnSe-like, lower mode) and hetero (CdSe-like, upper mode) environments. Under pressure, the Cd–Se bonds stiffen up faster ($d{f}_{i}^{*}/dlnV$ is large) than the Zn–Se ones ($d{f}_{i}^{*}/dlnV$ is small), with concomitant impact on the Zn–Se Raman shifts, being large for the upper mode and small for the lower one, meaning that the Zn–Se gap widens. In this line the Ga-P doublet of GaAs_1−x_P_x_ is also expected to widen—though to a less extent since the $d{f}_{i}^{*}/dlnV$-contrast is less in GaAs_1−x_P_x_ than in Zn_1−x_Cd_x_Se^24^. The trend line is actually there, as revealed by a careful examination of existing ab initio data^21^. The Be–Se (Zn_1−x_Be_x_Se) and Zn–S (ZnSe_1−x_S_x_) closures can be explained in the same way, which solves issue (i).

Generally, the frequency gap between the two Be–Se TO sub-modes of Zn_1−x_Be_x_Se is stable throughout the x-domain (within ~ 10%), that is the reason why the closure occurs around the same critical pressure ${P}_{c}$ at any x value. The gap within the Zn–S gap doublet of ZnSe_0.68_S_0.32_^22^ is roughly one-third the Be–Se one in Zn_1−x_Be_x_Se^21^; at the same time Zn–S rigidifies under pressure at a slower rate than Be–Se does, also by about one-third (Fig. 1)^24^. Hence, the Zn–S (small gap, low rate) and Be–Se (large gap, high rate) doublets close around the same ${P}_{c}$, by chance falling close to ${P}_{ZnSe}$^23^. This resolves issue (iii).

Remaining issues in the closure case relate to the (ii) Raman extinction of the lower mode and to (iv) its phonon freezing (i) on crossing the upper one at (${\omega }_{c}$,${P}_{c}$). A common origin is discussed below by using the well documented Be–Se doublet of Zn_1−x_Be_x_Se as a case study (Supplementary Section II).

Feature (ii) reveals that the lower TO mode actually “feels” the upper one when forced into its proximity by pressure. This suggests some coupling, i.e., a mechanical one then, owing to the purely-mechanical nature of the TOs. In this case one would a priori expect the repulsion of the two TOs in their tight-coupling at the resonance. However, this contradicts experimental findings demonstrating that the crossing, within experimental resolution, actually occurs (Fig. S11). In principle, the crossing makes sense only if the two TOs do not “see” each other, and hence are not coupled. This opposes to our basic premise.

The contradiction is removed by considering a model of two coupled but damped harmonic (mass + spring) 1D-oscillators with identical masses but different force constants standing for the Be–Se bonds vibrating in homo (BeSe-like, lower mode) and hetero (ZnSe-like, upper mode) environments. A recent TO-like version of such model^26^ is generalized to LOs in this work (Supplementary Section II). A pivotal feature in this model is the so-called exceptional point characterized by exact screening of the mechanical coupling by overdamping right at the resonance (${\omega }_{c},{P}_{c}$)—corresponding to a perfect tuning of the bare-uncoupled TO oscillators. Such screening leads, in fact, to a virtual decoupling of the TOs, so that an actual crossing is allowed—in reference to (i).

The picture which emerges is that the mechanical coupling between the two TOs is progressively undermined by the increasing damping of the lower mode on approach to the upper one—testified by (ii), until the as-overdamped system of coupled TOs resonantly locks into its exceptional point from ${P}_{c}$ onwards. In fact, no crossing can occur except in this point: the prevalence of either the mechanical coupling or the overdamping at the resonance leads to TO-anticrossing (Supplementary Section II)^26^.

The unique coupled-overdamped normal mode at the exceptional point, abbreviated exceptional mode below, manifests a compromise between the distinct symmetric and antisymmetric normal modes of the coupled system formed at the resonance in absence of damping. As the latter refer to in-phase and out-of-phase motions of oscillators—with equal magnitude, respectively, only one oscillator can be active in the exceptional mode, the other one has to be passive, being merely involved as a frozen/inert body—as sketched out in Fig. 1c (Supplementary Section II). This nicely resonates with the ab initio insight (iv). As such, the exceptional mode vibrates at nearly the same frequency as the active oscillator, hence assigned as the upper Be–Se mode (Fig. 1c). As for the passive oscillator, by freezing it becomes Raman inactive: the Raman effect results from modulation of the electronic susceptibility by a bond vibration, absent in this case. This is consistent with the lower mode suffering a Raman extinction from ${P}_{c}$ onwards (Fig. 1c)—referring to (ii).

In brief, the (Raman extinction, phonon-freezing, exceptional mode) triptych is consistent and outlines an appropriate framework to explain all pending issues (i–iv).

Retrospectively the above situation can be viewed as a damped variant of the non-linear 1D-lattice dynamics studied in a pioneering numerical experiment done on an extended chain of similar (in masses and spring constants) undamped oscillators^36^, and Ref.^1^ therein. In our case, the pointedly introduced anharmonicity—in reference to the mechanical coupling between the two TOs—fails to generate the chaotic dynamics observed by the cited authors, because on reaching the resonance at (${\omega }_{c}$,${P}_{c}$) the coupled system becomes overdamped and locks into its exceptional mode.

## Conclusion

**Figure 3:**
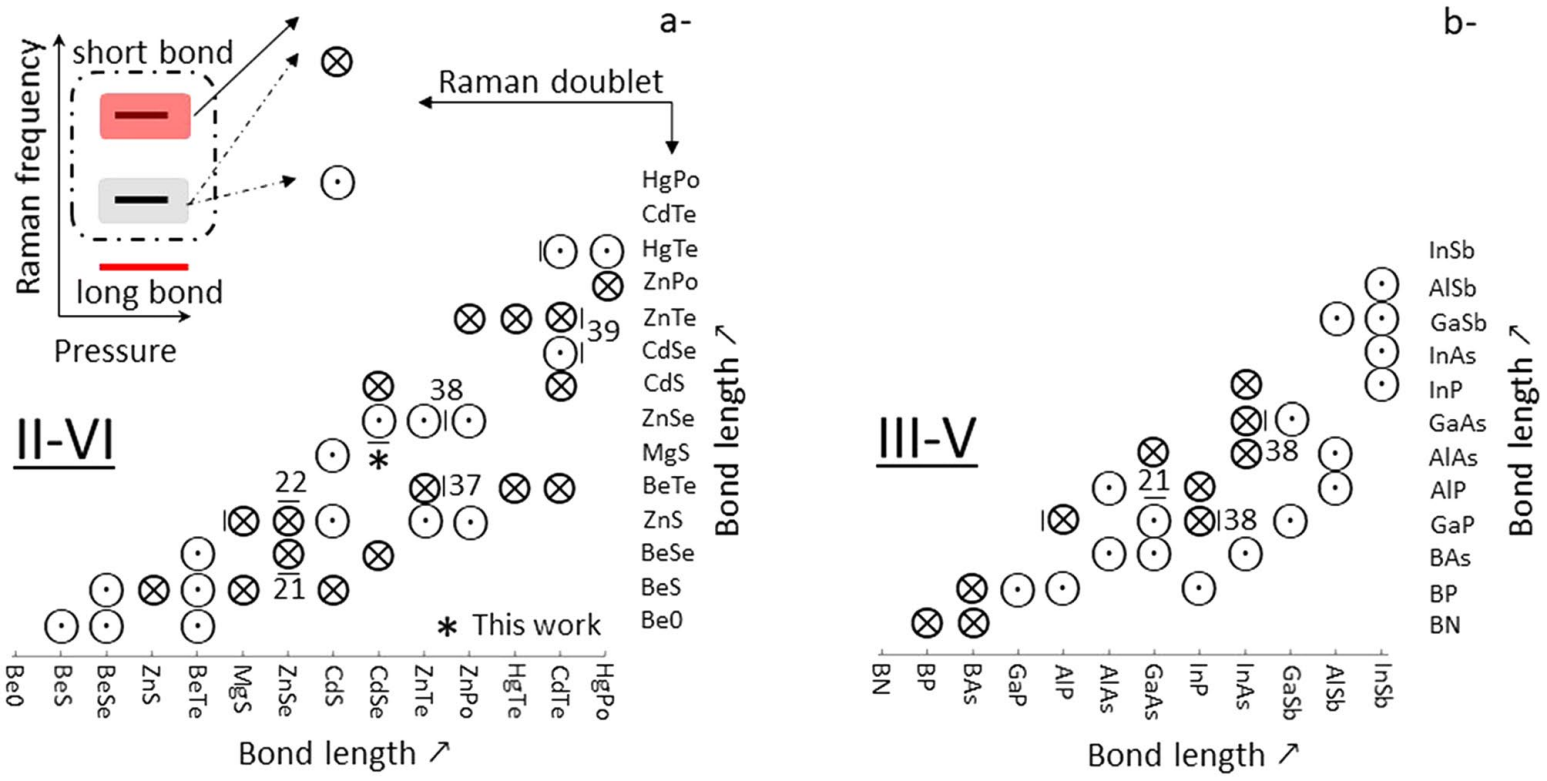
Partitions of II–VI and III–V semiconductor mixed crystals based on the pressure dependence of the Raman “percolation” doublet. (**a**) II–VI partition. (**b**) III–V partition. For each system the percolation-type Raman doublet refers to the short species that vibrates at high frequency with a distinction between homo (bottom) and hetero (top) environments, as sketched out. The pressure-induced closure/opening of a Raman doublet is inferred from the volume dependence of the bond ionicities given in Ref.^24^. Left bars refer to potentially problematic systems in which the short bond involves the large/heavy substituent. Alternative bars equipped with relevant references identify systems in which the percolation doublet has been evidenced (right bars) and further studied under pressure (upward or downward bars).

Summarizing, this work reveals a partition between A_1−x_B_x_C zincblende mixed crystals depending on whether they engage their pressure-induced structural transition with/without a rigid in-chain-backbone of self-connected bonds of the short species—when this is minor in the crystal (at least up to ~ 50 at.% in the case of Zn_1−x_Be_x_Se—Supplementary Section II). A decisive test is the closure/opening of the related percolation-type Raman doublet under pressure. The closure/opening is explained around the notion of a phonon exceptional point being achieved/avoided depending on the pressure dependence of the mechanical properties of the AC- and BC-like host media behind the Raman doublet. Such partitions for the common II–VI and III–V mixed crystals, compiled on the basis of the theoretical estimates of bond length and volume dependence of bond ionicity provided in Ref.^24^ (along the same line as discussed in this work), are shown in Fig. 3 including references to relevant works on II–VI^21,22,37–39^ and III–V^21,38^ systems.

We foresee potential applications in the closure case. The pressure can be tuned to release or inhibit the vibration along the backbone, offering an effective on/off phononic switch at the unusual mesoscopic scale. Additional flexibility arises in that the backbone can be continuous or segmented depending on whether the short bonds percolate or not. Generally, this is interesting in view to reduce the thermal conductivity. Besides, the pressure-induced freezing of part of the short/stiff bonds (the self-connected ones) vibrating at high frequency dramatically impacts the oscillator strength awarded to the related/upper $\Gamma$-like LO mode (Supplementary Section II). As the latter dominates the heat dissipation process of photoelectrons in zincblende semiconductors^40^, the pressure emerges as a possible means to play with the thermalization rate of electrons in mixed-semiconductor-based photovoltaic devices.

## Methods

This Section provides detail for replication and interpretation of the reported data. Additional experimental insights gained by nuclear magnetic resonance, high-pressure X-ray diffraction and high-pressure Raman scattering, together with statistical issues related to bond percolation phenomena as well as theoretical ones concerning coupled systems of damped harmonic 1D-oscillators—applied to Zn_0.48_Be_0.52_Se in this case—are reported as Supplementary Information (Sect. II).

### Sample growth and preparation

The used Zn_1−x_Cd_x_Se and Zn_1−x_Be_x_Se samples were grown by using the Bridgman method^41^ as large high-quality single crystals—as the ZnSe_1−x_S_x_ systems completing the current ZnSe-based series—and prepared for investigations as cylinders 3 mm in height (the as-grown crystals being 5–6 cm in length) and 8 mm in diameter. The purity of the ZnSe and CdSe compounds and of the Be material used to prepare the mixtures were 5 N (99.9995) and 2 N (99.5), respectively. The composition was determined by energy dispersive X-ray spectroscopy analysis. The composition gradient along the growth axis is negligible (less than 0.5 at.% Cd) for the considered length of the investigated samples. The particular Zn_0.83_Cd_0.17_Se and Zn_0.48_Be_0.52_Se crystals studied in the body of the manuscript and in the Supplementary Section II exhibit a pure zincblende structure at ambient pressure testified by X-ray diffraction (see Ref.^19^ for Cd_0.17_Zn_0.83_Se) and are characterized by a trend towards clustering (based on the current nuclear resonance magnetic measurements) and by a quasi random Zn ↔ Be substitution, respectively. Corresponding references for Zn_0.48_Be_0.52_Se are given in the course of the discussion.

### High-pressure Raman measurements

High-pressure unpolarized near-forward and backward Raman spectra are taken on Zn_0.83_Cd_0.17_Se by inserting, together with ruby chips used for pressure calibration (via the fluorescence linear scale^42^), a ~ 35 $\mathrm{\mu m}$-thick single crystal with parallel (110)-oriented faces obtained by cleavage inside a stainless-steel gasket preindented to 60 $\mathrm{\mu m}$ and drilled by spark-erosion to ~ 250 $\mathrm{\mu m}$, placed between the large diamonds (with a 400 $\mathrm{\mu m}$ culet) of a membrane Chervin type diamond anvil cell^43^. Methanol/ethanol/distilled-water (16:3:1), that remains hydrostatic up to ~ 10.5 GPa^44^, i.e., slightly beneath the zincblende → rock-salt pressure transition of the studied crystal (~ 12 GPa), is used as the pressure transmitting medium. Similar high-pressure Raman measurements performed with Zn_0.48_Be_0.52_Se are detailed as Supplementary Information (Sect. II).

The phonon-polaritons are detected by adopting the (nearly) perfect forward scattering geometry in which the incident laser beam (with wavevector ${\overrightarrow{k}}_{i}$) enters the rear of the crystal at nearly normal incidence and the scattered light (the wavevector is ${\overrightarrow{k}}_{s}$) is detected in front along the same direction. However, the zero value of the scattering angle $\theta$ = (${\overrightarrow{k}}_{i},{\overrightarrow{k}}_{s}$) inside the crystal cannot be achieved experimentally. The limiting factor is the numerical aperture of the lens used to collect the scattered light. Outside the crystal, the detected light fits into a pencil-like cone with half top angle $\alpha$ smaller than 4°. When brought back to a unidirectional beam, this corresponds to an average deviation by $\stackrel{-}{\alpha }$ ~ 0.4° from the normal to the crystal face (by averaging over $\mathrm{sin}\alpha \times d\alpha$). The angle for the corresponding scattered beam inside the crystal is scaled down to ~ 2.7 by the refractive index of the crystal, as measured by spectroscopic ellipsometry for the used green and blue laser lines at ambient pressure. With this, the minimal achievable $\stackrel{-}{\theta }$ value for the (average) scattering angle inside the crystal falls down to ${\stackrel{-}{\theta }}_{min}$ ~ 0.15°.

The dispersion and Raman intensities of the PP modes, including their TO and LO asymptotes, are obtained by using the same generic formula of the multi-mode Raman cross-section as in Ref.^19^. Detail concerning the pressure dependence of various input parameters coming into this formula is given as Supplementary Information (Sect. I—Zn_1−x_Cd_x_Se and Sect. II—Zn_1−x_Be_x_Se). The relevant $\stackrel{-}{\theta }$ value *per* PP Raman spectrum is estimated theoretically, *i.e*., via a fine tuning until the experimental “$\omega$ vs. $q$” Raman scan line derived from the wavevector conservation law that governs the Raman scattering process (i.e., ${\overrightarrow{k}}_{i}-{\overrightarrow{k}}_{s}=\overrightarrow{q}$, once expressed in its $\stackrel{-}{\theta }$-dependence using the relevant values of the refractive index for the incident and scattered lights) intercepts the “$\omega$ vs. $q$” PP dispersion right at the experimentally observed PP Raman frequencies. The as-obtained $\stackrel{-}{\theta }$ values at intermediate (~ 5 GPa) and maximum (~ 9 GPa) pressures (see Fig. 2) fall close to ${\stackrel{-}{\theta }}_{min}$, indicating that a nearly perfect forward scattering geometry is achieved experimentally.

A crucial ingredient in the calculation of the high pressure PP Raman cross-section is the dispersion of the refractive index of the crystal around the used laser lines at a given pressure. An experimental insight is a difficult task. A rough estimate, sufficient for our use, is obtained by translating as a whole the dispersion of the Zn_0.83_Cd_0.17_Se refractive index measured by ellipsometry at ambient pressure using a large crystal piece. The translation is guided by the pressure-induced step increase in the optical band gap of pure ZnSe, constituting a natural reference given the moderate Cd content. In doing so we rely on a theoretical prediction^45^ and proceed as earlier done with ZnSe_1−x_S_x_^22^.

The sample geometry is crucial. If the scattering setup does not strictly conform to normal incidence/detection onto/from (110)-oriented crystal faces—as if, e.g*.*, the laser beam impinges on a non-oriented edge of the tiny piece of single crystal inserted in the diamond anvil cell—the high-pressure Raman signal may be spoiled by the 2 × TA continuum, and may look extremely confusing. A decisive proof that the spurious 2 × TA continuum has been “killed”, meaning that all features in Fig. 2 can be safely discussed in terms of nominal one-phonon Raman-active modes, is the PP detection. Such modes can be readily identified experimentally based on their extreme sensitivity to change in the scattering angle and/or the laser line (Supplementary Section I, Fig. S6).

### Ab initio calculations

Ab initio calculation of the high-pressure TO (purely-mechanical) Raman spectrum of Zn_0.5_Cd_0.5_Se is done by implementing the formula given in Ref.^46^ within the Ab initio Modeling PROgram (AIMPRO) code^47,48^ operated in the density functional theory along the local density approximation for the exchange–correlation potential, using a 216-atom zincblende-type supercell optimized to a random Cd ↔ Zn substitution ($\kappa$ ~ 0, 50 at.% Cd) by simulated annealing. The retained criterion for randomness is that the distribution of Se-centered tetrahedrons with Cd/Zn atoms at the vertices forming the zincblende crystal (five in total) matches the corresponding $\kappa$-dependent Binomial Bernoulli distribution at the considered composition (extensive detail is given in Ref.^19^). The Raman calculations are done after full relaxation of the Zn_0.5_Cd_0.5_Se supercell (lattice constant and atom positions) using the basis functions and pseudopotentials detailed, together with accuracy issues, in Ref.^19^. In the high pressure study the third-order Birch–Murnaghan^49^ equation of state was used to determine the supercell volume. To test this approach, 10 GPa applied to a reference pure-ZnSe supercell produces an increase in the TO Raman frequency of 44.4 cm^−1^ in reasonable agreement with the experimental value^50^ of ~ 50.0 cm^−1^. The distribution of bond lengths and/or the phonon density of states (PhDOS) of Zn_1−x_Cd_x_Se and/or Zn_1−x_Be_x_Se depending on composition and/or pressure are generated on the same basis, to complete an ab initio insight into the lattice dynamics and/or the underlying lattice relaxation of such systems (Supplementary Section I, Figs. S9 and S10).

## Supplementary information

Supplementary Information.

## Data Availability

All data regarding the work presented here, including the Matlab routines for contour modeling of various (PP, TO, LO) multi-mode Raman cross sections and for statistical insight into the bond percolation phenomena in large-size random/clustered zincblende/wurtzite-type supercells are available upon reasonable request to the corresponding author.
